# Rhizosphere Microbiome Recruited from a Suppressive Compost Improves Plant Fitness and Increases Protection against Vascular Wilt Pathogens of Tomato

**DOI:** 10.3389/fpls.2017.02022

**Published:** 2017-11-29

**Authors:** Anastasis Antoniou, Maria-Dimitra Tsolakidou, Ioannis A. Stringlis, Iakovos S. Pantelides

**Affiliations:** ^1^Department of Agricultural Sciences, Biotechnology and Food Science, Cyprus University of Technology, Limassol, Cyprus; ^2^Plant-Microbe Interactions, Department of Biology, Faculty of Science, Utrecht University, Utrecht, Netherlands

**Keywords:** compost, rhizosphere, *Fusarium oxysporum*, *Verticillium dahliae*, disease suppression, plant growth promotion, microbiome

## Abstract

Suppressive composts represent a sustainable approach to combat soilborne plant pathogens and an alternative to the ineffective chemical fungicides used against those. Nevertheless, suppressiveness to plant pathogens and reliability of composts are often inconsistent with unpredictable effects. While suppressiveness is usually attributed to the compost’s microorganisms, the mechanisms governing microbial recruitment by the roots and the composition of selected microbial communities are not fully elucidated. Herein, the purpose of the study was to evaluate the impact of a compost on tomato plant growth and its suppressiveness against *Fusarium oxysporum* f. sp. *lycopersici* (Foxl) and *Verticillium dahliae* (Vd). First, growth parameters of tomato plants grown in sterile peat-based substrates including 20 and 30% sterile compost (80P/20C-ST and 70P/30C-ST) or non-sterile compost (80P/20C and 70P/30C) were evaluated in a growth room experiment. Plant height, total leaf surface, and fresh and dry weight of plants grown in the non-sterile compost mixes were increased compared to the plants grown in the sterile compost substrates, indicating the plant growth promoting activity of the compost’s microorganisms. Subsequently, compost’s suppressiveness against Foxl and Vd was evaluated with pathogenicity experiments on tomato plants grown in 70P/30C-ST and 70P/30C substrates. Disease intensity was significantly less in plants grown in the non-sterile compost than in those grown in the sterile compost substrate; AUDPC was 2.3- and 1.4-fold less for Foxl and Vd, respectively. Moreover, fungal quantification *in planta* demonstrated reduced colonization in plants grown in the non-sterile mixture. To further investigate these findings, we characterized the culturable microbiome attracted by the roots compared to the unplanted compost. Bacteria and fungi isolated from unplanted compost and the rhizosphere of plants were sequence-identified. Community-level analysis revealed differential microbial communities between the compost and the rhizosphere, suggesting a clear effect of the plant in the microbiome assembly. Proteobacteria and Actinobacteria were highly enriched in the rhizosphere whereas Firmicutes were strongly represented in both compartments with *Bacillus* being the most abundant species. Our results shed light on the composition of a microbial consortium that could protect plants against the wilt pathogens of tomato and improve plant overall health.

## Introduction

Being sessile organisms, plants are confined in one place and cannot escape any adverse conditions of their environment, including interactions with microbes of different lifestyles. The interactions between plants and microbes may occur at different sites in the aerial parts of the plants and in the rhizosphere. Rhizosphere can be defined as the soil layer adjacent to roots that is highly affected by root exudation (Hiltner, 1904; Hartmann et al., 2008). Plant rhizodeposits can be diverse including amino acids, organic acids, polysaccharides, and proteins and constitute a valuable food source. That makes exudates important determinants of microbial composition in the rhizosphere, due to nutrient competition, and drivers of the increased microbial activity in the root–soil interface compared to the usually starved soil (Bais et al., 2006; Raaijmakers et al., 2009; Mendes et al., 2013). The mesmerizing diversity of microbes settling down in the rhizosphere are termed as root microbiome while their interaction with roots can result in positive or negative outputs for plant fitness (Berendsen et al., 2012; Hacquard et al., 2015). Recently, increasing evidence suggests that the microbial communities dominating the rhizosphere are influenced by the host genotype (Bulgarelli et al., 2012, 2013; Badri et al., 2013). It appears that plants actively select and determine the composition of the root microbiome by releasing compounds in the rhizosphere that selectively stimulate microorganisms promoting plant growth and health or repress organisms that are deleterious to the plants (Doornbos et al., 2012). Thus, root microbiome is a subset of a more diverse microbial community recruited from the surrounding bulk soil.

The involvement of the root microbiome on plant health becomes more evident in disease suppressive soils. In these soils, a plant is unlikely to become infected by a soilborne pathogen even when the pathogen is present and favored by the plant. The phenomenon of disease suppression is well-known and is associated with the indigenous microbiota and activity (Weller et al., 2002; Haas et al., 2005). However, the mechanisms underlying this phenomenon are not fully understood and have become a major focus of current research. The role of the microorganisms as primary factors in disease suppression can be highlighted by the fact that most suppressive soils lose their suppressive activity when pasteurized or sterilized (Weller et al., 2002; Haas et al., 2005). The ability of soils to suppress soilborne pathogens has been considered to be either general or specific. General suppression is attributed to the total microbiome of the soil (Berendsen et al., 2012; Cha et al., 2016) and is not transferable between soils (Cook and Rovira, 1976; Rovira and Wildermuth, 1981). Pathogens are inhibited due to the direct antagonistic and competitive activities of the soil microbiota and suppressiveness is enhanced by the addition of organic matter, composts, and other agronomic practices that increase the total microbial activity in the soil (Hoitink and Boehm, 1999; Weller et al., 2002). It offers only a basal protection against a broad range of pathogens and the suppression is not attributed to a specific microorganism (Weller et al., 2002). In contrast, specific suppression is due to the activities of specific microorganisms that act against a particular pathogen and is more effective than the general suppression (Weller et al., 2002; Berendsen et al., 2012; Cha et al., 2016). It is characterized by transferability of the suppressiveness by introducing an inoculum of 0.1–10% of suppressive soil into a conducive soil (Cook and Rovira, 1976; Weller et al., 2002). Regardless of the mechanisms that influence the interactions between the beneficial microorganisms and the pathogens, the amendments of soil with composts reshape the microbial community structure and new equilibria are established (Hadar and Papadopoulou, 2012).

Composts are organic soil amendments with known suppressive properties against various soilborne pathogens, including *Fusarium* and *Verticillium* species (Borrero et al., 2004; Bonanomi et al., 2007; Malandraki et al., 2008; Papasotiriou et al., 2013). Compost suppressiveness has been attributed to biotic and/or abiotic factors (Noble and Coventry, 2005) and a substantial number of studies demonstrated that, as in soils, the disease suppressive effects of composts are lost after sterilization or pasteurization indicating the microbial population of the compost as the main factor responsible for suppressiveness (Gorodecki and Hadar, 1990; Cotxarrera et al., 2002; Reuveni et al., 2002; Tilston et al., 2002; Bonanomi et al., 2010; Mendes et al., 2011; Papasotiriou et al., 2013). A considerable amount of research addressed the microbiological nature of compost suppressiveness by isolating and identifying many different antagonistic bacteria and fungi responsible for conferring disease suppression (Gorodecki and Hadar, 1990; Cotxarrera et al., 2002; Reuveni et al., 2002; Tilston et al., 2002). Several bacterial and fungal genera have been identified as biological control agents in compost-amended substrates using cultivation-based techniques and have been used as inoculants to improve the consistency of disease control using composts (Kwok et al., 1987; Nakasaki et al., 1998; De Ceuster and Hoitink, 1999; Hoitink et al., 2001; Trillas et al., 2006; Pugliese et al., 2011). However, the investigation of the microbial consortia of suppressive composts rather than single microbial species would provide valuable information to our understanding of the compost microbiome and how its members interact with plants (Hadar and Papadopoulou, 2012). Additionally, identification of the microbial diversity present in a suppressive compost that is actively attracted by plant roots could allow the development and employment of microbial mixtures that could function more efficiently toward plant-growth promotion and pathogen suppression compared to individual bacterial/fungal strains (Stockwell et al., 2011; Sundaramoorthy et al., 2012; Sarma et al., 2015; Hu et al., 2017; Xiong et al., 2017; Weidner et al., 2017). Such approaches are relatively recent and have not been investigated widely.

The use of suppressive composts is now an established approach for the biological control of soilborne plant pathogens (Hoitink and Fahy, 1986; Noble and Coventry, 2005; Hadar, 2011). Suppressive composts are of particular interest especially for the management of vascular wilt pathogens that are difficult to control. Fungal wilt pathogens *Fusarium oxysporum* and *Verticillium dahliae* are among the most devastating soilborne pathogens, causing many economically important diseases in annual and perennial crops worldwide. They cause a wide range of symptoms depending on the host, the virulence of the pathogen, and the environmental conditions. Symptoms include epinasty, chlorosis, partial or complete wilting, stunting, and ultimately death (Fradin and Thomma, 2006; Michielse and Rep, 2009). Controlling fungal wilt pathogens is difficult for a number of reasons as they can infect a wide range of plants, including weeds and volunteer plants, and consequently primary inoculum sources are maintained nearby the fields (Yadeta and Thomma, 2013). Currently, there are no effective treatments that can cure plants once they get infected. Additionally, both *F. oxysporum* and *V. dahliae* produce persistent resting structures that survive in the soil for many years in the absence of a susceptible host. Thus far, resistant cultivars, biological control agents, and organic soil amendments are the most effective strategies to control fungal wilt diseases (Tsuda et al., 2001; Suárez-Estrella et al., 2007; Markakis et al., 2008, 2016; Angelopoulou et al., 2014; Kefalogianni et al., 2017).

The first objective of this study was to investigate the potential suppressive effects of a compost on the major wilt pathogens of tomato, *V. dahliae* (Vd) and *F. oxysporum* f. sp. *lycopersici* (Foxl), and to evaluate the impact of the compost on tomato plant growth. The compost is produced in Cyprus exclusively from recycled plant origin material of Cyprus. The second objective of the current study was to identify the culturable bacteria and fungi of the compost and to provide insights into the composition of the compost’s microbial community recruited by tomato roots based on rRNA sequencing. In this context, we aimed to identify bacteria and fungi associated with the rhizosphere of tomato plants which in a next step could be tested for their potential to improve plant health and plant productivity.

## Materials and Methods

### Compost

The compost used in this study is produced in Cyprus (My Green Cycle, Premier Shukuroglou Cyprus Ltd.) exclusively from recycled plant origin material of Cyprus (e.g., lawn, garden clippings, trees, vines, etc.). Sample of the compost was air-dried to determine physicochemical characteristics before conducting the experiments. Analysis of the compost was performed at a commercial laboratory (Aristos Loucaides Chemical Laboratory Ltd., Cyprus) accredited in accordance with the recognized International Standard ISO/IEC 17025:2005. Physical–chemical properties of the compost are summarized in **Table 1**.

**Table 1:** Physicochemical characteristics of the compost.

Compost characteristics	Value	Unit
pH	7.65	–
Electrical conductivity	3.75	mS/cm
Organic matter	49.9	% dry basis
Ash	50.1	% dry basis
NO^3-^ – N	69	mg kg^-1^
C/N	23	–
Phosphorus	233	mg kg^-1^
Potassium	0.790	% dry basis
CaCO_3_	1.2	% dry basis
Copper	3	mg kg^-1^
Manganese	78	mg kg^-1^
Zinc	68	mg kg^-1^
Iron	23	mg kg^-1^
Sodium	2,557	mg kg^-1^
Chlorides	6,498	mg kg^-1^
Arsenic	4.16	mg kg^-1^
Mercury	<0.40	mg kg^-1^
Lead	5.89	mg kg^-1^
Nickel	20.6	mg kg^-1^
Chromium	27.4	mg kg^-1^
Cadmium	<0.40	mg kg^-1^
Cobalt	<0.40	mg kg^-1^
Boron	9.13	mg kg^-1^


### Preparation of Potting Mixes

For the bioassays with tomato plants four potting substrates were prepared. Amended substrates consisted of: (a) 80% sterile peat and 20% compost (80P/20C), (b) 80% sterile peat and 20% sterile compost (80P/20C-ST), (c) 70% sterile peat and 30% compost (70P/30C), and (d) 70% sterile peat and 30% sterile compost (70P/30C-ST). Mixes percentages were prepared as volume per volume (v/v). Peat and substrate mixes containing sterile compost were used after steam-sterilization (100°C for 1 h in 3 consecutive days) to eliminate all microorganisms present in these components. Substrates were kept in a laminar flow unit at room temperature in between sterilizations. CaCO_3_ was added to peat (7 g l^-1^) to increase the pH at ideal range (6.0–6.5) that maximizes availability of plant nutrients. Plant available nutrients were measured for 70P/30C and 70P/30C-ST substrates to test whether compost sterilization affected nutrients availability (Supplementary Table S2). No significant differences were observed in plant-available nutrients of the substrates. However, mineral fertilizers were added to the potting mixes to equilibrate nutrient levels in all treatments. A commercial fertilizer containing N (5.9%${NO}_{3}^{-}$ - N, 3.9%${NH}_{4}^{+}$ - N, 10.2% urea-N), P_2_O_5_ (20%), K_2_O (20%), Fe ethylene diamine tetraacetic acid (Fe-EDTA; 0.1%), Zn-EDTA (0.05%), Mn-EDTA (0.05%), Cu-EDTA (0.05%), B (0.02%), and Mo (0.0005%) was applied at a concentration of 1 g l^-1^. The same batch of peat was used in all bioassays (Plantobast Peat, Plantaflor).

### Evaluation of Tomato Plant Growth with Compost

Tomato (*Solanum lycopersicum*) cv. Ailsa Craig (Thompson & Morgan Ltd.) was used in the experiments. Seeds were sown directly into 8 cm diameter pots, each containing approximately 500 cm^3^ potting mix. The pots were placed in a controlled-environment growth room [dimensions: 500 cm (L) × 340 cm (W) × 270 cm (H)] at 25°C with a 16-h photoperiod and 65–70% RH, with a photosynthetic photon flux density of 450 μmol m^-2^ s^-1^ at pot height. Plants were arranged in rows and columns in a completely randomized design, at a plant density of 40 plants m^-2^, with 10 replicates (plants) per treatment and were rotated within the growth room every second day before watering. The plants were watered individually every second day with equal volume of water. Plant growth was evaluated 30 days after seedling emergence (DASE) by recording shoot height in centimeters (cm), measuring shoot fresh and dry weight with a precision analytical balance and calculating total leaf area. For the latter, all individual leaves of each tomato plant were cut with their petiole from the stem, mounted on a white paper, and scanned. The scanned pictures were subsequently analyzed by ImageJ software^1^ that uses a threshold-based pixel count measurement to calculate leaf area of plants (Sack and Frole, 2006; Schneider et al., 2012; Barros et al., 2017; Klein et al., 2017). The experiment was repeated three times.

### Evaluation of Compost Effectiveness against Fungal Wilt Pathogens of Tomato

Compost suppressiveness against the fungal wilt pathogens of tomato was evaluated by pathogenicity experiments performed on tomato plants, at the four-leaf stage. Tomato plants were grown in 70P/30C and 70P/30C-ST substrates under the same conditions and arrangement as described above. The fungal isolates of Vd (race 1 isolate 70V, Pantelides et al., 2010) and Foxl (race 1 isolate Fol004, Rep et al., 2005) were cryopreserved as an aqueous 20% glycerol suspension at -80°C and before being used they were transferred to potato dextrose agar (PDA, Merck) and incubated at 25°C for 5 days. For the pathogenicity experiments, conidia were prepared by transferring pieces from the growing edge of the fungal colony of each pathogen in sucrose sodium nitrate (SSN) (Sinha and Wood, 1968) in Erlenmeyer flasks and incubated in an orbital shaker at 150 rpm in the dark for 5 days. Vd was grown at 22°C and Foxl at 25°C. Suspensions were centrifuged at 10,000 × *g*, 12°C for 10 min, and resuspended in sterile distilled water (SDW) before being applied to the plants. Plants were inoculated with Vd or Foxl by root drenching with 10 ml conidial suspension containing 10^7^ conidia ml^-1^ SDW. Control plants were mock-inoculated with 10 ml SDW. Disease severity was calculated by the number of leaves showing typical symptoms as a percentage of the total number of leaves of each plant. Symptoms were periodically recorded for 31 and 24 days post inoculation (dpi) for Vd and Foxl, respectively. Disease ratings were plotted over time to generate disease progress curves. The area under the disease progress curve (AUDPC) was calculated by the trapezoidal integration method (Campbell and Madden, 1990). The experiment was repeated three times with 10 replicates per treatment.

### DNA Extraction and Fungal Biomass Quantification *in Planta*

The level of fungal colonization in the vascular tissues of tomato plants was assessed by real-time quantitative PCR (qPCR). Tomato plants were grown in 70P/30C and 70P/30C-ST substrates and inoculated with Vd or Foxl at the four-leaf stage as described previously. Fifteen plants from each treatment were harvested at 5-day intervals from 5 to 25 dpi. For each sampled plant the leaves were discarded, the stem was cut at soil level and ground to a fine powder using mortar and pestle with liquid nitrogen. Total DNA was extracted according to Dellaporta et al. (1983) with small modifications (20 min incubation time at 65°C, DNA pellet redissolved in TE with 200 μg RNase ml^-1^) and quantified by spectrophotometry.

Quantification assays with real-time qPCR were performed using the primer pairs ITS1-F: 5′-AAAGTTTTAATGGTTCGCTAAGA-3′, ST-VE1-R5′-CTTGGTCATTTAGAGGAAGTAA-3′ (Ellendorff et al., 2009) targeting the internal transcribed spacer (ITS) region of the ribosomal DNA of Vd, and sp1-2f: 5′-GCTGGCGGATCTGACACTGT-3′, sp1-2pr: 5′-TTTCGTACTTGCCAGGTTG-3′ (Inami et al., 2010) targeting the rDNA-intergenic spacer of Foxl. For sample equilibration, the *S. lycopersicum* b-tubulin gene was targeted using the primer pair LeTUB-F: 5′-GATTTGCCCACTAACCTCTCGT-3′ and LeTUB-R: 5′-ACCTCCTTTGTGCTCATCTTACCC-3′ (Pantelides et al., 2010). Real-time qPCR conditions consisted of an initial 95°C denaturation step for 3 min, followed by 40 cycles of denaturation at 95°C for 3 s, and annealing/extension at 60°C for 30 s. Primer specificity and formation of primer–dimers were monitored by dissociation curve analysis. For the quantification of the pathogens, a standard curve was constructed for each pathogen with 10-fold dilutions of the corresponding PCR product. The fungal biomass quantification experiments were repeated three times.

### Isolation and Enumeration of Bacteria and Fungi of Compost

Six compost samples of about 25 g were collected from unplanted pots and transferred in Stomacher bags with 225 ml Maximum Recovery Diluent (MRD; Oxoid, United Kingdom) to obtain a 10-fold dilution. After a 2-min homogenization in a stomacher (Stomacher 400 Circulator, Seward, United Kingdom) successive 10-fold dilutions were done as required and 100 μl aliquots were plated in triplicate on agar plates. The total number of cultivable bacteria and fungi were determined as colony forming units (CFUs) on tryptic soy agar (TSA; Oxoid, United Kingdom) and on sabouraud dextrose agar (SDA) amended with chloramphenicol (0.1 g l^-1^), respectively. Agar plates were incubated at 25°C for 48 and 72 h for bacteria and fungi, respectively, prior to colony counting. Microbial populations were expressed in terms of cfu g^-1^. Colonies were purified and all isolates from three biological samples were preserved as glycerol stock at -80°C.

### Isolation of Microorganisms from the Rhizosphere

Tomato plants were grown as described previously in 70P/30C substrate mix for 30 DASE. For the isolation of rhizospheric bacteria and fungi, 15 plants (3 replicates × 5 plants) were carefully uprooted from the substrate and loosely adhering compost mixture was gently removed from the roots by shaking. Roots with the remaining adhering rhizosphere soil were placed in sterile MRD (Oxoid, United Kingdom) in a shaking incubator (120 rev min^-1^) for 30 min at 25°C. Serial 10-fold dilutions were prepared with the wash solution and spread on TSA and SDA/chloramphenicol plates. Agar plates were incubated at 25°C for 48 and 72 h for bacteria and fungi, respectively, and after colony purifications, isolates from three replicates were kept as a glycerol stock at -80°C.

### Microbial DNA Extraction, PCR Amplification, and Sequencing

Bacterial DNA was extracted from each isolate using a rapid boiling method. In brief, three to four colonies were homogenized in 20 μl lysis buffer (0.25% SDS, 0.05 N NaOH), vortexed, and incubated for 10 min at 95°C. Samples were centrifuged for 1 min and vortexed vigorously after the addition of 180 μl of 10 mM Tris–HCl (pH 8.5). After centrifugation at 16,000 rpm min^-1^ at 4°C, supernatant was transferred in fresh tube and stored at -20°C until use. Fungal DNA extraction was performed according to Cary et al. (2009). Piece from the growing edge of the fungal colony (∼300 mg of mycelia) of each isolate was transferred in Eppendorf tube and homogenized with 700 μl LETS buffer (0.1 M LiCl: 20 mM EDTA pH 8, 10 mM Tris–HCl pH 8, 0.5% SDS) with disposable pestle. Samples were vortexed briefly, 700 μl of phenol:chloroform:isoamyl alcohol (25:24:1) was added and kept on the bench for 5 min. After centrifugation for 10 min at 16,000 rpm min^-1^ at 4°C, supernatant was transferred in fresh tube and DNA was precipitated with 1 ml ethanol (90%). DNA was pelleted with 10 min centrifugation at 13,000 rpm min^-1^ at 4°C and washed with 70% ethanol before resuspension in SDW. DNA samples were stored at -20°C until use. PCR amplifications were carried out using 10 ng of template DNA and 12 pmol of each primer in 30 μl reaction mixture containing 1× Kapa Taq buffer, 0.2 mM dNTPs, 1.5 mM MgCl2, 1 U Kapa Taq (Kapa Biosystems, Wilmington, MA, United States). Fungal rDNA ITS region was amplified using ITS 1 (5′-TCCGTAGGTGAACCTGCGG-3′) and ITS 4 primers (5′-TCCTCCGCTTATTGATATGA-3′) (White et al., 1990) and bacterial 16S rDNA was amplified with primers 27F (5′-AGAGTTTGATCMTGGCTCAG-3′) and 907R (5′-CCGTCAATTCMTTTRAGTTT-3′) (Lane, 1991). Amplifications were performed in a C100^®^ Thermal Cycler (Bio-Rad, Hercules, CA, Unites States) and the cycle parameters were: 1 cycle at 95°C for 3 min; 35 cycles of 94°C for 30 s, 58°C for 30 s and elongation at 72°C for 1 min; followed by 1 cycle at 72°C for 10 min. Specific amplification of a single PCR product with the expected length was confirmed with electrophoresis on 1% agarose gel containing SYBR safe DNA gel stain (Invitrogen, Carlsbad, CA, United States). PCR products were purified with the MinElute PCR Purification Kit (Qiagen, Hilden, Germany) and sequencing reactions were carried out with a BigDye Terminator v3.1 Cycle Sequencing Kit (Applied Biosystems, Foster City, CA, United States) as per the manufacturer’s protocol. Sequencing products were purified to eliminate excess fluorescent dyes by NaOAc/EtOH precipitation and then analyzed by using ABI 3130 Genetic Analyzer (Applied Biosystems). The sequencing output was analyzed using the Sequencing Analysis Software version 5.4 (Applied Biosystems) and sequences were compared and identified by a database similarity search in the GENBANK Collection using the BLAST algorithm^2^.

### Statistical Analyses

Data were analyzed by one-way ANOVA (analysis of variance, Tukey’s test with *P* ≤ 0.05 or two-way ANOVA, Sidak’s test with *P* ≤ 0.05) using GraphPad Prism 7 for Windows (GraphPad Software, La Jolla, CA, United States^3^). R language (R 2017,^4^ version 3.3.3) was run from R-studio environment (version 1.0.136) and was used for the culturable microbiome analyses. Both relative abundance of a cultured microbe, normalized by the total number of isolated microbes per sample replicate (total sum normalization), and β-diversity [principal coordinates analysis (PCoA) using Bray–Curtis dissimilarities] was calculated in the “phyloseq” package (version 1.19.1; McMurdie et al., 2013). Package ggplot2 (version 2.2.1) was used for the visualization of β-diversity (Wickham, 2009).

## Results

### Total Viable Counts of Culturable Bacteria and Fungi

**FIGURE 1:**
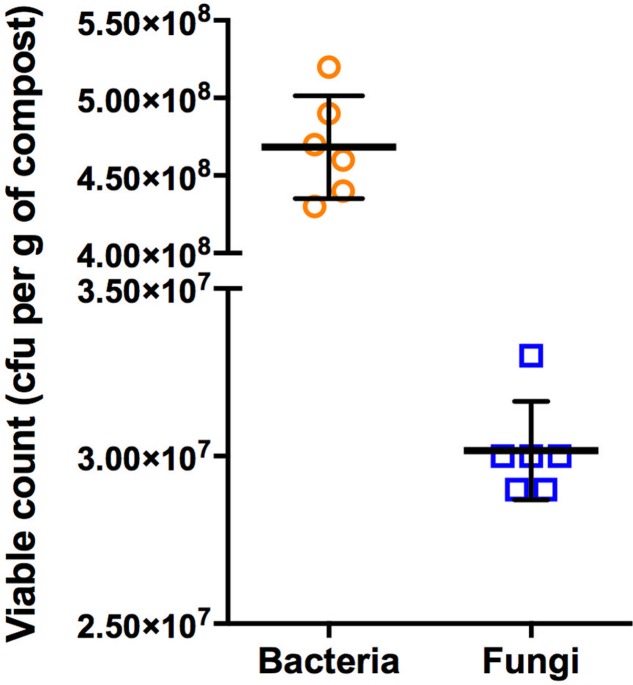
Scatter plot and mean ± SD of CFU of bacteria and fungi after 48 and 72 h incubation at 25°C, respectively. The values are plotted as CFU per gram of compost. Circles and squares represent raw data of six replicates for bacteria and fungi, respectively.

The enumeration of culturable bacteria and fungi was performed on unplanted compost samples in order to determine the microbiological load of the compost. For these experiments, different culture media were initially evaluated for their performance under the experimental conditions. TSA and SDA were selected for their ability to support the greatest number of isolates for bacteria and fungi, respectively. These media are commonly used for the isolation of microorganisms from soils and composts (Ellis et al., 2003; Vieira and Nahas, 2005; Bai et al., 2015; Stefani et al., 2015; Haas et al., 2016; Muñoz et al., 2017). The microbial analyses indicated that the total number of bacteria in the compost was greater than the number of fungi. In particular, the total viable counts of bacteria in the bulk compost were, on average, 15.5-fold higher in comparison with the fungi (**Figure 1**). This set of experiments were performed on the same batch of compost used throughout all the experiments of this study.

### Changes in Growth Parameters of Tomato Plants Grown in Sterile and Non-sterile Compost Substrate

**FIGURE 2:**
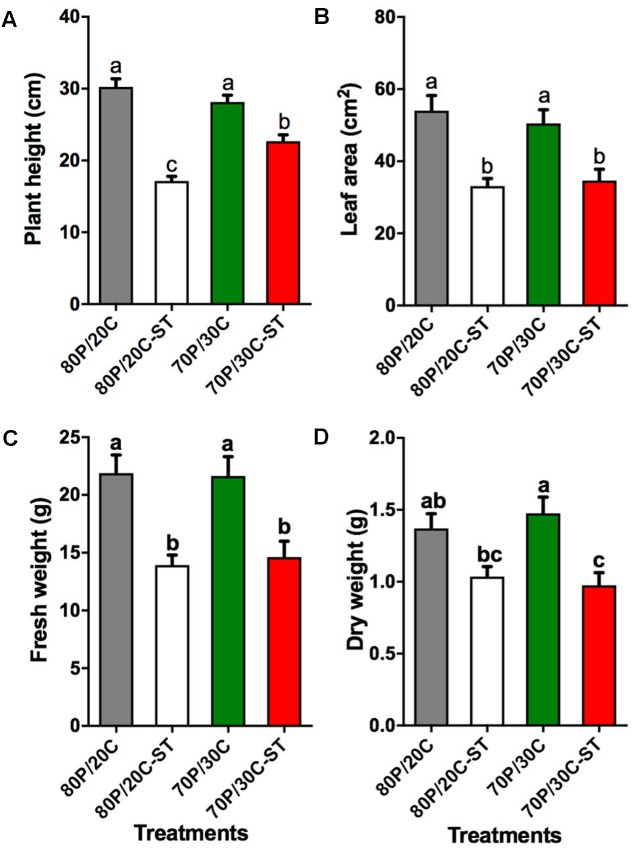
Effect of potting mixes consisting of peat and compost on plant growth of tomato plants. Data are means of 10 biological replicates. Error bars represent SE. Different letters represent statistically significant differences between treatments (one-way ANOVA, Tukey’s test; *P* < 0.05). **(A)** Plant height (in cm). **(B)** Leaf area (in cm^2^). **(C)** Fresh weight of plants (in g). **(D)** Dry weight (in g).

In a first step to characterize the effect of the compost microbiome on plant growth, tomato plants were grown in sterile or non-sterile potting mixes consisting of peat and compost. The growth measurements were performed 30 DASE, to decrease the potential influence of flowering on growth parameters. Plants grown in both non-sterile substrates visibly stimulated increases in plant height (**Figure 2A**), leaf area (**Figure 2B**), and fresh weight (**Figure 2C**) relative to the plants grown in the sterilized substrates. However, no significant differences were observed between plants cultivated in the growing media with different compost content except for plant height; plants grown in 70P/30C-ST were significantly taller compared to plants grown in 80P/20C-ST. When plants were grown in the 70P/30C mix an increase in dry weight was observed, although the extent of the increase was not significant compared to the 80P/20C mix (**Figure 2D**). For these reasons, all the following experiments were performed by using the 30% compost mixes (70P/30C and 70P/30C-ST).

### Microorganisms Are Involved in the Suppressiveness of the Compost against the Fungal Wilt Pathogens of Tomato

**FIGURE 3:**
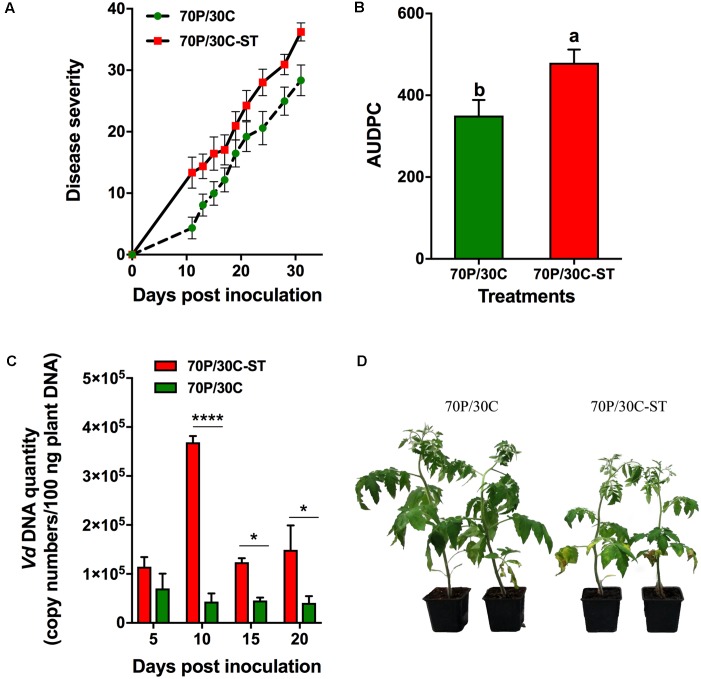
Verticillium wilt disease severity on tomato plants inoculated with *Verticillium dahliae* and fungal biomass quantification *in planta*. **(A)** Disease severity at each observation was calculated by the number of leaves that showed wilting as a percentage of the total number of leaves of each plant. Each treatment consisted of 10 plants and the experiment was repeated three times with similar results. Vertical bars indicate the standard error of mean. **(B)** Disease ratings were plotted over time to generate disease progress curves; subsequently, the area under the disease progress curve (AUDPC) was calculated by the trapezoidal integration method. Columns with different letters are statistically significantly different according to Tukey’s multiple range test at *P* < 0.05. **(C)** Quantification of fungal biomass *in planta* was performed by real-time qPCR using total plant DNA isolated from the aboveground parts of plants, sampled at 5, 10, 15, and 20 days post-inoculation (dpi). Data are means of 15 plants and error bars indicate SE. Statistically significant differences are indicated as ^∗^*P* < 0.05 and ^∗∗∗∗^*P* < 0.0001 (two-way ANOVA, Sidak’s test). **(D)** Verticillium wilt symptoms on tomato plants grown in the non-sterilized (Left) and in the sterile mix (Right) at 15 dpi.

**FIGURE 4:**
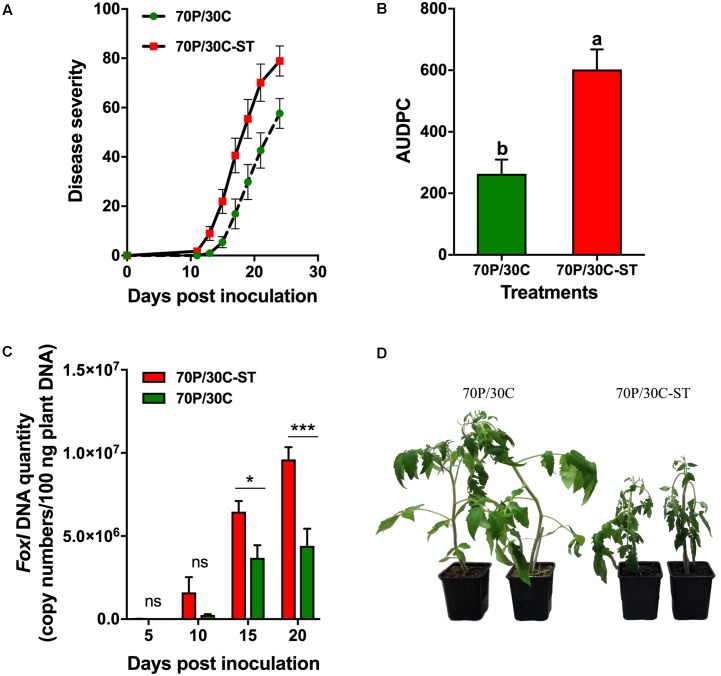
Fusarium wilt disease severity on tomato plants inoculated with *F. oxysporum* f. sp. *lycopersici* and fungal biomass quantification *in planta*. **(A)** Disease severity at each observation was calculated by the number of leaves that showed wilting as a percentage of the total number of leaves of each plant. Each treatment consisted of 10 plants and the experiment was repeated three times with similar results. Vertical bars indicate the standard error of mean. **(B)** Disease ratings were plotted over time to generate disease progress curves; subsequently, the AUDPC was calculated by the trapezoidal integration method. Columns with different letters are statistically significantly different according to Tukey’s multiple range test at *P* < 0.05. **(C)** Quantification of fungal biomass *in planta* was performed by real-time qPCR using total plant DNA isolated from the aboveground parts of plants, sampled at 5, 10, 15, and 20 dpi. Data are means of 15 plants and error bars indicate SE. Statistically significant differences are indicated as ^∗^*P* < 0.05 and ^∗∗∗∗^*P* < 0.0001 (two-way ANOVA, Sidak’s test). **(D)** Fusarium wilt symptoms on tomato plants grown in the non-sterilized (Left) and in the sterile mix (Right) at 15 dpi.

The potential suppressive effects of the compost were assessed in pathogenicity experiments using tomato plants against Vd and Foxl. Plant responses were evaluated by recording the wilting symptoms induced by the pathogens. In these experiments, tomato plants were grown in 70P/30C and 70P/30C-ST substrates to assess whether the microbial consortium of the compost is associated with suppressiveness. The first Vd symptoms appeared in the form of wilting and yellowing especially on older leaves at 11 dpi and were recorded until 31 dpi (**Figure 3D**). Disease (in the form of wilting followed by yellowing and necrosis of leaves) progressed more rapidly in the plants grown in the sterile compost mix, while the plants in the non-sterile substrate showed less prominent symptoms and slower disease development (**Figure 3A**). At 31 dpi, disease incidence (% plants with symptoms) in the 70P/30C plants was 96.7% (data not shown) and disease severity 28.3% (**Figure 3A**), whereas in the 70P/30C-ST plants disease incidence was 100% (data not shown) and disease severity 36.2% (**Figure 3A**). The AUDPC marker over 31 days of disease progress was 27% higher in the 70P/30C-ST plants than in the 70P/30C plants (**Figure 3B**). Regarding bioassays of tomato plants with Foxl, the first disease symptoms were observed at 11 and 13 dpi on 70P/30C-ST and 70P/30C plants, respectively. Symptoms of the disease (wilting, yellowing, defoliation, and necrosis) were recorded until 24 dpi (**Figure 4D**). Similarly, the plants grown in the non-sterile compost mix exhibited slower disease development and less severe symptoms compared to the plants grown in the sterile mix (**Figure 4A**). At 24 dpi, disease incidence was 100% in both treatments (data not shown) and disease severity was 78.9% in 70P/30C-ST and 57.7% in 70P/30C plants (**Figure 4A**). AUDPC over 24 days of disease progress was 56.3% higher in the 70P/30C-ST plants compared to the 70P/30C plants (**Figure 4B**).

### Fungal Biomass Quantification *in Planta* Reveals Reduced Colonization in Plants Grown in the Non-sterile Compost Substrate

Data obtained from the pathogenicity tests showed that plants grown in the non-sterile mix were less affected by the pathogens compared to the plants grown in the sterile mix. To determine whether the reduced disease severity of the 70P/30C plants is associated with a reduction in the amount of the pathogen in the vascular tissues of the plants, the level of fungal colonization was assessed by real-time qPCR after inoculation of plants grown in the sterile and non-sterile mixes with the wilt pathogens. Analysis of real-time qPCR showed that by 5 dpi, Vd was already present in the vascular tissues of both 70P/30C-ST and 70P/30C plants. Five days later at 10 dpi the amount of the pathogen in the 70P/30C-ST plants was 8.5 times greater than in the 70P/30C plants. At 15 dpi the amount of the pathogen in the 70P/30C-ST plants declined three times while in the 70P/30C plants did not change significantly. However, the relative amount of pathogen DNA was 1.8 times higher in 70P/30C-ST than 70P/30C plants. At 20 dpi, the same colonization pattern as at 15 dpi was observed with the amount of the pathogen being 3.7 times greater in the 70P/30C-ST compared to the 70P/30C plants. It is worth mentioning that the levels of Vd were significantly lower in the 70P/30C than in the 70P/30C-ST plants at all sampling time points, except 5 dpi (**Figure 3C**). Similarly, real-time qPCR analysis showed that Foxl was first detected in the vascular tissues of plant at 10 dpi. The pathogen presence increased steadily until 20 dpi in both treatments with the amount of pathogen being significantly lower in the 70P/30C than in the 70P/30C-ST plants at 15 and 20 dpi, ranging from 1.5 to 1.8 times less in the plants grown in the non-sterile vs. the sterile substrate (**Figure 4C**).

### Culture-Dependent Analysis of Bacterial and Fungal Population in the Compost and Rhizosphere of Tomato Plants

**FIGURE 5:**
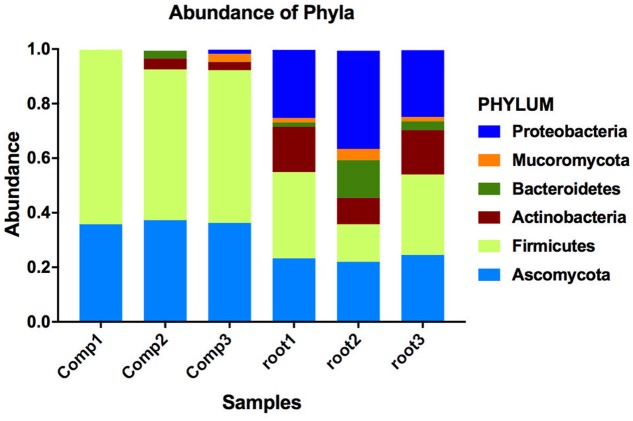
Distribution of phyla is altered between the two compartments. Bar plot showing the relative abundance of phyla where the isolated bacteria and fungi are classified. Relative abundance was calculated for each culturable microbe dividing the times it was isolated in each sample with the total number of isolated microbes in this sample (total sum normalization). Different colors correspond to different phyla and segment sizes of stacked bars are proportional to the relative abundance of the phyla (comp1, comp2, comp3 = compost; root1, root2, root3 = rhizosphere).

**FIGURE 6:**
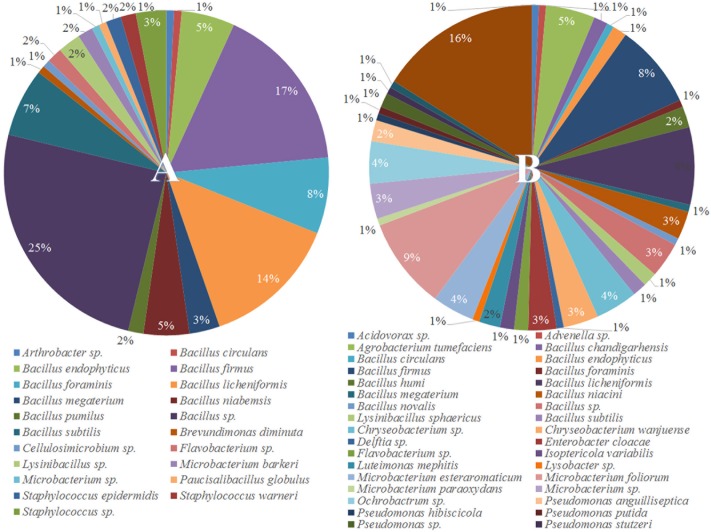
Pie chart representation of the total cultured (relative abundance) bacteria **(A)** isolated from the unplanted compost and **(B)** the rhizosphere of tomato plants.

**FIGURE 7:**
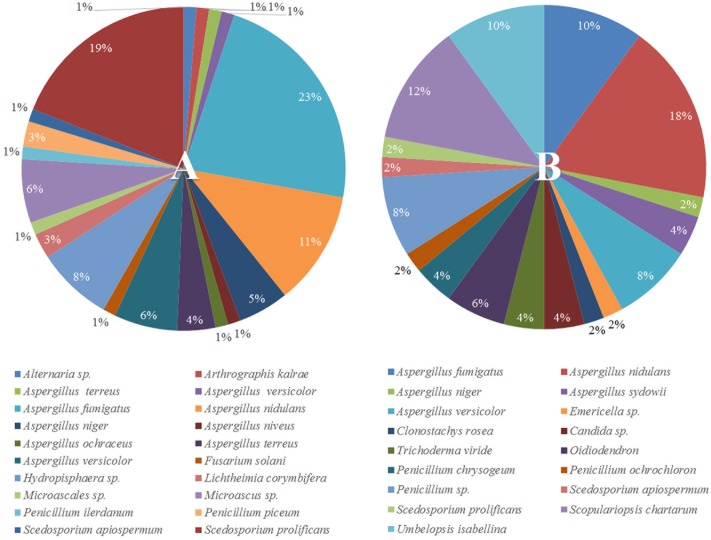
Pie chart representation of the total cultured (relative abundance) fungi **(A)** isolated from the unplanted compost and **(B)** the rhizosphere of tomato plants.

To understand why plants growing in non-sterilized compost mixes grow better and are more resistant against soilborne pathogens, we aimed to characterize the culturable microbiome attracted in tomato roots and its composition compared to compost that remained unplanted. A total of 132 bacterial isolates and 79 fungal isolates were cultured from the unplanted compost in three independent experiments. Regarding rhizosphere samples, 143 bacterial and 50 fungal isolates were cultured from three independent experiments. Through 16S and ITS rDNA sequence identification, we demonstrated that these microorganisms could be classified into 72 distinct species, 47 bacterial and 25 fungal (Supplementary Table S1). Actinobacteria, Bacteroidetes, Firmicutes, Proteobacteria, Ascomycota, and Mucoromycota were the six dominant phyla accounting for 100% of the total microbial taxa, despite their differential abundance between unplanted compost and rhizosphere samples (**Figure 5**). Regarding bacteria, Firmicutes were much more abundant than other phyla in different compost samples, accounting for 55.2–64% of the total taxa (**Figure 5**) and included 15 species and 124 isolates (**Figure 6A**). Species in phylum Firmicutes included *Bacillus circulans*, *Bacillus endophyticus*, *Bacillus firmus*, *Bacillus foraminis*, *Bacillus licheniformis*, *Bacillus megaterium*, *Bacillus niabemsis*, *Bacillus pumilus*, *Bacillus* sp., *Bacillus subtilis*, *Lysinibacillus* sp., *Paucisalibacillus globulus*, *Staphylococcus epidermidis*, *Staphylococcus warneri*, and *Staphylococcus* sp. (**Figure 6A**). Actinobacteria was the second most abundant phylum in compost samples accounting for 0–4% of the total taxa (**Figure 5**) and contained five isolates and three genera including *Arthrobacter* sp., *Cellulosimicrobium* sp., and *Microbacterium* sp. (**Figure 6A**). The phyla Bacteroidetes and Proteobacteria had the lowest abundance accounting for 0–2.9 and 0–1.5% of the total taxa (**Figure 5**) and included only *Flavobacterium* sp. and *Brevundimonas diminuta*, respectively (**Figure 6A**). Regarding fungi, Ascomycota was the most dominant phylum of compost samples, accounting for 35.8–37.3% of the total taxa (**Figure 5**) including 19 species and 77 isolates (**Figure 7A**). Species in phylum Ascomycota included *Alternaria* sp., *Arthrographis kalrae*, *Aspergillus terreus*, *Aspergillus versicolor*, *Aspergillus fumigatus*, *Aspergillus nidulans*, *Aspergillus niger*, *Aspergillus niveus*, *Aspergillus ochraceus*, *Aspergillus terreus*, *Fusarium solani*, *Hydropisphaera* sp., *Microascales* sp., *Microascus* sp., *Penicillium ilerdanum*, *Penicillium piceum*, *Scedosporium apiospermum*, and *Scedosporium prolificans* (**Figure 7A**). Mucoromycota had low abundance accounting for 0–3% of the total taxa (**Figure 5**) and included only *Lichtheimia corymbifera* (**Figure 7A**).

The dominant phyla described in unplanted compost were the most prevalent phyla in rhizosphere samples as well. However, tomato roots displayed an apparent effect on their abundance. Regarding bacteria, Proteobacteria were highly enriched in the rhizosphere, with abundance 57.0 ± 7.5 times higher than that of unplanted compost, accounting for 24.5–36% of the total taxa (**Figure 5**) and contained 56 isolates and 15 species including *Acidovorax* sp., *Advenella* sp., *Agrobacterium tumefaciens*, *Delftia* sp., *Enterobacter cloacae*, *Luteimonas mephitis*, *Lysobacter* sp., *Ochrobactrum* sp., *Pseudomonas anguilliseptica*, *Pseudomonas hibiscicola*, *Pseudomonas putida*, *Pseudomonas* sp., *Pseudomonas stutzeri*, *Sinorhizobium adhaerens*, and *Stenotrophomonas maltophilia* (**Figure 6B**). The abundance of Actinobacteria in the rhizosphere was 6.1 ± 1.0 times of that in unplanted compost, accounting 9.7–16.6% of the total taxa (**Figure 5**) including 27 isolates and 4 species including *Isoptericola variabilis*, *Microbacterium esteraromaticum*, *Microbacterium foliorum*, and *Microbacterium paraoxydans* (**Figure 6B**). The abundance of Bacteroidetes was 6.4 ± 3.9 times higher in the rhizosphere than that of bulk compost, accounting for 1.6–13.8% of the total taxa (**Figure 5**) and including 13 isolates and 3 species including *Flavobacterium* sp., *Chryseobacterium* sp., and *Chryseobacterium wanjuense* (**Figure 6B**). However, the abundance of Firmicutes in the rhizospheric samples was lower than in the unplanted compost for about 2.3 ± 0.1 times, accounting for 13.8–31.6% of the total taxa (**Figure 5**) and contained 47 isolates and 11 species including *Bacillus chandigarhensis*, *B. circulans*, *B. endophyticus*, *B. firmus*, *B. foraminis*, *Bacillus humi*, *B. licheniformis*, *B. megaterium*, *Bacillus niacin*, *Bacillus novalis*, and *B. subtilis* (**Figure 6B**). Regarding fungi, Mucoromycota were slightly enriched in the rhizosphere, with abundance 2.4 ± 0.8 times higher than that of unplanted compost, accounting for 1.6–4.1% of the total taxa (**Figure 5**) and containing five isolates *Umbelopsis isabellina* (**Figure 7B**). The abundance of Ascomycota in the rhizosphere was slightly lower than in the unplanted compost for about 1.6 ± 0.1 times, accounting for 22–24.5% of the total taxa (**Figure 5**) and contained 45 isolates and 14 species including *A. fumigatus*, *A. nidulans*, *A. niger*, *Aspergillus sydowii*, *A. versicolor*, *Clonostachys rosea*, *Candida* sp., *Trichoderma viride*, *Oidiodendron* sp., *Penicillium chrysogeum*, *Penicillium ochrochloron*, *S. apiospermum*, *S. prolificans*, and *Scopulariopsis chartarum* (**Figure 7B**).

**FIGURE 8:**
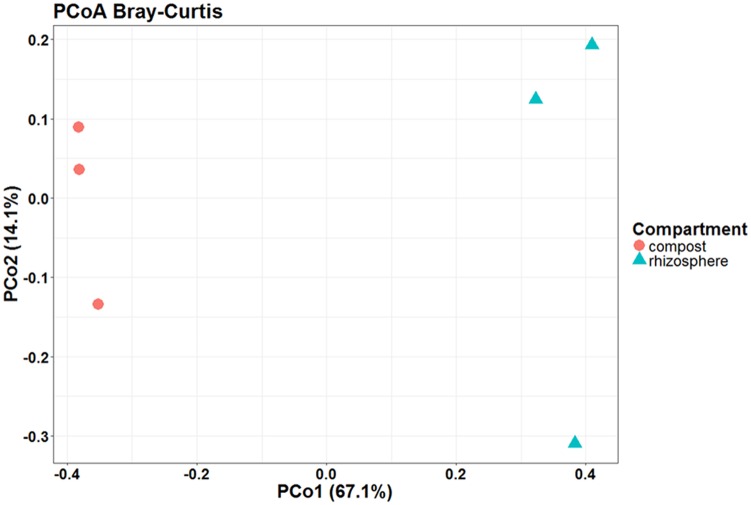
Presence of the plant causes changes in the microbial communities of the compost. Principal coordinates analysis (PCoA) with Bray–Curtis dissimilarity distances shows that compost (red circle) and rhizosphere (blue triangle) are clearly separated, suggesting that the compartment explains the highest percentage of the variation between the isolated microbial communities. Number of circles and triangles with same color represent the number of samples per compartment.

Principal coordinates analysis based on Bray–Curtis dissimilarity confirmed the differences observed between the isolated microbial communities of tomato roots and unplanted compost (**Figure 8**). In that case, the compartment could explain by 67.1% of the total variance the differences observed in the relative abundance of the OTUs, as identified in this study in the unplanted compost (comp1, comp2, comp3) and rhizosphere samples (root1, root2, root3).

## Discussion

Nowadays, a lot of emphasis is given on finding sustainable methods to combat plant diseases, aiming to eliminate the negative impact of currently used approaches on public health and the environment. Application of composts and other organic amendments has long been proposed as an alternative strategy to control plant diseases, especially those caused by soilborne pathogens that are difficult to control with other conventional strategies, such as synthetic fungicides and resistant cultivars. There is a substantial number of studies demonstrating the effective control of soilborne pathogens by the application of composts and the disease suppression conferred by organic amendments and composts has been systematically reviewed. Bonanomi et al. (2007) analyzed 250 articles with 2,423 experimental case studies focusing on the suppressiveness of different organic amendments and found that compost was the most suppressive material, with more than 50% of cases showing effective disease control. Noble (2011) reviewed 79 container and 59 open-field experiments and found the application of compost showing a disease suppressive effect in ∼75% of cases. Noble and Coventry (2005) also reviewed disease suppression by composts in several cases and concluded that the suppressive effects of compost on soilborne diseases are more consistent in container-based studies compared to results obtained in the field. In another comprehensive study, Termorshuizen et al. (2006) found that compost application had suppressive effects in 54% of all cases. Although the phenomenon of suppressiveness is well reported, the ability of composts to control plant diseases is often inconsistent with unpredictable effects, limiting the practical use and commercialization of these materials. There has been a considerable amount of research into the phenomenon of suppressiveness suggesting that there is no single factor conferring this property to soils or composts. However, it is well documented that suppressiveness is conferred by the microbial populations occupying these ecosystems as it is eliminated by heat treatment or by γ-irradiation (Weller et al., 2002; Haas et al., 2005; Noble and Coventry, 2005; Malandraki et al., 2008; Bonanomi et al., 2010; Mendes et al., 2011).

In the present study, our primary goal was to investigate the potential suppressive properties of a compost against the main fungal wilt pathogens of tomato and to evaluate the effects of compost application on plant growth of tomato. Disease suppressiveness was studied in compost-amended peat under controlled environmental conditions and although pathogens were inoculated at high densities the tomato plants grown in the compost showed slower disease development and less severe symptoms compared to the control plants for both pathogens. Microbial nature of the suppressiveness was demonstrated by the application of sterilized compost in control plants resulting in loss of the suppressive activity (**Figures 3**, **4**). Additionally, application of the compost visibly and quantitatively stimulated increases in plant height, leaf area, and fresh and dry weight of tomato plants compared to the controls (**Figure 2**), suggesting that the microbial community of the compost promotes plant growth. In order to clarify whether the less prominent wilting symptoms on plants is the result of disease suppression preventing pathogen colonization or a direct promotion of plant growth regardless of the presence or absence of pathogen, fungal biomass was quantified *in planta.* Our results showed that both pathogens were found significantly lower in plants grown in the compost corroborating the results of the pathogenicity experiments. Our findings are in line with previous container-based studies demonstrating a suppressive effect of plant origin composts on wilts caused by Foxl (Raj and Kapoor, 1997; Trillas et al., 2002; Borrero et al., 2004) and Vd on tomato plants (Borrero et al., 2002) or other hosts (LaMondia et al., 1999; Paplomatas et al., 2005; Termorshuizen et al., 2006).

Rhizosphere has been characterized as a dynamic ecological niche where soil microorganisms interact with plant roots (Bais et al., 2006). Plants influence and shape the microbial community structure through their root architecture, the release of nutrients, and by changing the pH of soil (Bais et al., 2006; Badri and Vivanco, 2009). Exudation of organic compounds by the root leads to enhanced abundance and activity of selected microorganisms in the rhizosphere as compared to bulk soil; phenomenon described as the “rhizosphere effect” (Berg et al., 2006; Berg and Smalla, 2009; Bakker et al., 2013). It has been estimated that a substantial proportion of the photosynthetically fixed carbon is exuded from roots where it is metabolized by the microbial community in the rhizosphere, driving multitrophic interactions in this highly competitive living environment (Badri and Vivanco, 2009; Neumann et al., 2009). Root exudates can also influence rhizosphere interactions serving as signals that initiate symbiotic relationships that help plants to tolerate abiotic stresses, stimulate growth, or induce plant defenses against pathogens and insects (Mendes et al., 2011; Pieterse et al., 2014; Pérez-Jaramillo et al., 2016) or mediate negative interactions including association with parasitic plants, pathogenic microbes, and invertebrate herbivores (Badri and Vivanco, 2009). The variation in the rhizospheric microbial community in different types of soils is attributed to the soil background (Schlaeppi et al., 2013) thus the soil is a determining factor in the formation of rhizosphere microbiome (Garbeva et al., 2008; Lundberg et al., 2012). As in our study the compost was proven to be suppressive against the vascular wilt diseases of tomato, our secondary goal was to understand whether this suppressiveness was due to differences in the microbial composition of the rhizosphere and the unplanted compost. Metagenomic methodologies allow the analysis and description of the entire microbial communities either culturable or unculturable within complex environmental samples. Isolation of the culturable fraction of the microbial community allows the characterization of functions attributed to specific member(s) of the microbiome and the construction of synthetic communities that can be utilized in improving plant performance under different stresses (Castrillo et al., 2017). In the current study, we managed to isolate and characterize the microbes present in the suppressive compost and those enriched in the rhizosphere of tomato plants growing in this compost. We observed that bacterial and fungal community composition in genus level was highly dependent on the compartment with rhizosphere samples clearly differentiating from the unplanted compost (**Figure 8**). These results are in accordance with previous studies demonstrating that plants attract or inhibit the growth of specific microorganisms through rhizodeposition and root exudation thereby leading to the formation of the rhizosphere microbiome (Bais et al., 2006; Lugtenberg and Kamilova, 2009). In our study, Firmicutes and Ascomycota dominate the compost while Actinobacteria, Proteobacteria, Bacteroidetes, and Mucoromycota are rarely isolated (**Figure 5**). The bacterial Phyla found in the studied compost are also reported in other works describing the dominant taxa in suppressive soils (Mendes et al., 2011; Rosenzweig et al., 2012; Cha et al., 2016). Significant changes were observed in the Phylum composition of the rhizosphere where Ascomycota and Firmicutes were reduced while Actinobacteria, Bacteroidetes, and Proteobacteria were increased. The most striking shifts were that of Proteobacteria that were enriched 57 times in the rhizosphere samples while Actinobacteria increased their incidence 6.1 times compared to the unplanted compost samples. In contrast, Firmicutes were reduced 2.3 times in the rhizosphere samples compared to unplanted compost (**Figure 5**). A number of studies focusing on the bacterial taxa inhabiting the rhizosphere of several hosts revealed that the microbial community is dominated mainly by Proteobacteria, Firmicutes, and Actinobacteria and to a lesser extent by Bacteroidetes and Acidobacteria (Deangelis et al., 2005; Teixeira et al., 2010; Uroz et al., 2010; Mendes et al., 2011; Weinert et al., 2011). The increased abundance of Proteobacteria and Actinobacteria in the roots could be associated with the enhanced growth and the elevated potential of tomato plants to defend against the soilborne pathogens tested here, making them pools of microbes with antimicrobial and growth promoting abilities that should be further explored.

In our study, out of the 48 different species isolated from the rhizosphere, 17 were also cultured from the unplanted compost whereas 31 were exclusively found in the rhizosphere samples (**Figures 6**, **7**). The most abundant bacterial species in both unplanted compost and rhizosphere samples belonged to *Bacillus* sp. (**Figure 6**). Bacilli are considered as typical soil bacteria that are associated with plant roots with well-established beneficial effects on plant growth and biocontrol (Kloepper et al., 2004; Compant et al., 2005). Previous studies on *Bacillus* strains revealed numerous beneficial effects such as promotion of seedling emergence, synthesis of cyclic lipopeptides and polyketides with distinct antimicrobial action, production of plant-growth-stimulating volatiles (2,3-butanediol) that can also initiate ISR, etc. (Koumoutsi et al., 2004; Ryu et al., 2004; Chen et al., 2006). Some of the species that were enriched in the rhizosphere have been reported in the literature to be beneficial for the plants either by producing secondary metabolites with antimicrobial activity or by inducing plant defense and growth promotion. *Microbacterium* spp. (16.8% of the rhizosphere bacteria) are widespread bacteria and can be isolated from various habitats including soil and plants and many members of the genus possess the ability to induce plant growth promotion (Sheng et al., 2009; Madhaiyan et al., 2010; Lin et al., 2012). *Stenotrophomonas maltophilia* (16.1% of the rhizosphere bacteria) is a cosmopolitan species that is found usually in soil and plants and is able to produce antimicrobial compounds that protect plants and factors that can promote plant growth (Ryan et al., 2009). *Chryseobacterium* spp. (7.7% of the rhizosphere bacteria) are commonly found in soil and water and some species can suppress plant diseases without suppressing the growth of beneficial bacteria (Krause et al., 2001; McSpadden Gardener and Weller, 2001). *Pseudomonas* spp. (5.6% of the rhizosphere bacteria) are ubiquitous bacteria in soils and well adapted to growing in the rhizosphere of plants (Weller, 2007). Pseudomonads are accountable for the suppressiveness of some soils against soilborne pathogens (Weller et al., 2002) including *F. oxysporum* (Peer et al., 1991; Leeman et al., 1995) and they possess many characteristics that make them appropriate as biocontrol agents and plant growth promoters (Weller, 1988). *E. cloacae* (2.8% of the rhizosphere bacteria) is commonly found on or in plants and different sources in the environment (e.g., water, soil) (Nishijima et al., 2004) and many members of the species are biological protectants that effectively suppress diseases caused by *Pythium*, *Sclerotinia*, and *Fusarium* (Hadar et al., 1983; Sneh et al., 1984; Nelson and Craft, 1991; Trutmann and Nelson, 1992). Regarding fungi, small numbers of *Clonostachys rosea* (2% of the rhizosphere fungi) and *T. viride* (4% of the rhizosphere fungi) were isolated from rhizosphere samples but were not detected in unplanted compost. *Trichoderma* and *Clonostachys* are two of the most common fungi in soil and certain fungi from these genera are currently used as biological control agents. Their biocontrol activity is the result of various mechanisms acting synergistically, including induced resistance in plants, competition for nutrient and space, production of cell wall degrading enzymes, or direct antagonism (Harman et al., 2004). Enrichment of the above-mentioned bacterial and fungal genera in the rhizosphere suggests that tomato plants actively select microbes from the compost, with well-described traits, that can explain the positive effects observed in tomato growth and the enhanced resistance against the tested soilborne pathogens.

Collectively, data presented herein support the notion that tomato plants can assemble diverse rhizosphere communities compared to the unplanted compost and recruit beneficial soil microorganisms for protection against fungal wilt infections. It is beyond the scope of this study to conclusively determine the microorganisms responsible for the suppressive phenotype of the compost, however, our results provide prior evidence on the composition of a microbial consortium that could efficiently protect plants and have a positive impact on plant overall health. Future studies need to address the same questions using a culture-independent approach to provide more insight into the diversity of microbes recruited in the rhizosphere that contribute in plant protection against fungal wilt pathogens. In our study, sterile peat was used as basic material to make combinations of compost for the plant cultivation. Thus, our results represent the first step toward the understanding of a complex phenomenon, and future experiments need to test whether compost incorporation in field soil would lead in increased plant protection, under field conditions, due to the recruitment of similar microbial communities in the rhizosphere. In addition, with this study we have been able to isolate a large collection of bacteria and fungi. This will allow us to construct synthetic microbial communities that could be used as inoculum to increase the consistency and efficiency of composts against soilborne pathogens and protect production systems where chemical pesticides and fertilizers have already failed.

## Author Contributions

IP conceived and designed the experiments. AA and M-DT performed the experiments. M-DT, IS, and IP analyzed the data. M-DT, IS, and IP wrote the paper. All authors have read and approved the manuscript.

## Conflict of Interest Statement

The authors declare that the research was conducted in the absence of any commercial or financial relationships that could be construed as a potential conflict of interest.

## Footnotes

1 https://imagej.nih.gov/ij/

2 http://blast.ncbi.nlm.nih.gov/Blast.cgi

3 www.graphpad.com

4 https://www.r-project.org
